# Serum untargeted lipidomics by UHPLC-ESI-HRMS aids the biomarker discovery of colorectal adenoma

**DOI:** 10.1186/s12885-022-09427-1

**Published:** 2022-03-24

**Authors:** Hailin Zhou, Yanying Nong, Yifan Zhu, Yunxiao Liang, Jiahao Zhang, Hongwei Chen, Pingchuan Zhu, Qisong Zhang

**Affiliations:** 1 Medical College of Guangxi University, Guangxi University, Nanning, Guangxi 530004 PR China; 2 Department of Gastroenterology, Ruikang Hospital Affilated to Guangxi University of Chinese Medicine, Nanning, Guangxi 530011 PR China; 3 Department of Gastroenterology, People’s Hospital of Guangxi Zhuang Autonomous Region, Nanning, Guangxi 530021 PR China; 4 State Key Laboratory for Conservation and Utilization of Subtropical Agro-Bioresources, Guangxi University, Nanning, Guangxi 530004 PR China

**Keywords:** Colorectal adenoma, Biomarkers, Serum lipidomics, Early screening, UHPLC-ESI-HRMS, Colorectal cancer

## Abstract

**Background:**

Colorectal adenoma (CA) is an important precancerous lesion and early screening target of colorectal cancer (CRC). Lipids with numerous physiological functions are proved to be involved in the development of CRC. However, there is no lipidomic study with large-scale serum samples on diagnostic biomarkers for CA.

**Methods:**

The serum lipidomics of CA patients (*n* = 50) and normal control (NR) (*n* = 50) was performed by ultra high performance liquid chromatography-high resolution mass spectrometry with electrospray ionization (UHPLC-ESI-HRMS). Univariate and multivariate statistical analyses were utilized to screen the differential lipids between groups, and combining the constituent ratio analysis and diagnostic efficiency evaluation by receiver operating characteristic (ROC) curve disclosed the potential mechanism and biomarkers for CA.

**Results:**

There were obvious differences in serum lipid profiles between CA and NR groups. Totally, 79 differential lipids were selected by criterion of *P* < 0.05 and fold change > 1.5 or < 0.67. Triacylglycerols (TAGs) and phosphatidylcholines (PCs) were the major differential lipids with ratio > 60%, indicating these two lipid metabolic pathways showed evident disequilibrium, which could contribute to CA formation. Of them, 12 differential lipids had good diagnostic ability as candidate biomarkers for CA (AUC ≥ 0.900) by ROC analysis.

**Conclusions:**

To our knowledge, this is the first attempt to profile serum lipidomics and explore lipid biomarkers of CA to help early screening of CRC. 12 differential lipids are obtained to act as potential diagnostic markers of CA. PCs and fatty acids were the main dysregulated biomarkers for CA in serum.

## Background

Colorectal cancer (CRC) is a significant public health problem and lethal disease, causing more than 900,000 deaths annually as a common malignant tumor worldwide [1]. It is reported that the high mortality rate of CRC is primarily due to the diagnosis and discovery of late-stage tumors. The early CRC stage has no specific symptoms, which generally leads to poor diagnostic effects and low detection rate. While the treatment effect in the middle and late stage is less favorable and along with many side effects [2]. The 5-year survival rate of CRC patients diagnosed at the early stage is about 90%, while it dramatically decreases to 14% for patients diagnosed with advanced-stage CRC [3]. In addition, nearly 90% of CRC evolves from colorectal adenoma (CA) [4]. The adenoma-carcinoma sequence is widely regarded as the main pathway for the formation and development of CRC currently [5], and most CA patients are associated with better treatment outcome and prognosis [6]. Thus, CA is an important target for early screening of CRC, and effective CA screening reduces the morbidity and mortality of CRC enormously. At present, the mainstays of CA screening methods including the fecal occult-blood test, stool DNA test, sigmoidoscopy, CT colonography, and optical colonoscopy. However, these methods also have apparent defects, such as poor performance for early diagnosis, high cost and technical requirements, and low patient compliance [6], which seriously limit their application in extensive screening of CA. Accordingly, it is vital to develop a minimally invasive diagnostic strategy with high performance to improve the early screening and prevention of CRC.

Lipids have emerged as important biomolecules involved in the numerous physiological processes of human that plays a diverse role in cell apoptosis, proliferation, signal transduction, and energy metabolism [7–10]. Growing evidence suggests that metabolic disorder of lipid is closely related to the progression of CRC disease [11–13]. As a key branch and advanced technique of metabolomics, lipidomics systematically and comprehensively reflects the changes in lipid profiles and related metabolic pathways within organisms under different physiological or pathological states [14]. Ultra high performance liquid chromatography tandem high resolution mass spectrometry with electrospray ionization (UHPLC-ESI-HRMS) has become the most prevalent analytical tool in lipidomics study due to its advantages of high selectivity, high sensitivity, and high throughput [15]. Currently, lipidomics has developed into a field with widespread application in biology, medicine, and chemistry science, because of its great potential in discovery of molecular mechanisms and biomarkers of diseases, and it has recently become a hotspot in omics research [16]. To date, several lipidomics studies on CRC have been reported. The lipidomics research found that lysophosphatidylcholines (LPCs) and phosphatidylcholines (PCs) are the most strongly related biomarkers of CRC formation [17, 18]. In plasma, ethanolamine plasmalogens and fatty acids (FAs) are considered as early diagnostic biomarkers of CRC [19]. Furthermore, triacylglycerols (TAGs) are found to be the main disturbed lipid markers of CRC progression [20, 21]. However, no study has been reported to explore the biomarkers of CA through serum lipidomics. Plasma untargeted LC-MS-based metabolomics is applied to investigate the potential mechanism of CA, indicating that L-tryptophan, L-proline, and lysoPC (C17:0) could be combined to serve as the biomarker to improve its diagnosis [22]. Studies suggest that total TAGs levels in serum or plasma are elevated may be associated with increased risk of CA [23]. According to some authors, the disruption of polyunsaturated fatty acids (PUFAs) is correlated with CA development [24, 25]. Therefore, the discovery of CA biomarkers based on serum lipidomics still needs to be studied, and which will be expected to solve the shortcomings of current screening methods for CA.

In this study, we performed a lipidomics study of serum samples from fifty CA patients and fifty healthy subjects by the UHPLC-ESI-HRMS technique. By compared with serum lipid profiles of CA and normal control (NR) groups, the differential lipids and potential mechanism of lipid metabolism pathways were explored by univariate and multivariate statistical analysis. Then, combined with receiver operating characteristic (ROC) curve analysis and trend change analysis of differential lipids, the potential lipid markers for CA diagnosis were evaluated and selected, which would provide a reference for early screening of CRC.

## Methods

### Chemicals and reagents

HPLC grade methanol, dichloromethane, isopropanol, acetonitrile, formic acid and ammonium formate were purchased from Merck & Co. (Billerica, MA, USA). Ultrapure water was prepared by a Millipore Milli-Q system (Billerica, MA, USA). Lipid standards including palmitoyl ethanolamide, palmitic acid, methyl palmitate, 2-arachidonoyl glycerol, and 4-dodecylbenzenesulfonic acid were obtained from Sigma-Aldrich (St Louis, MO, USA).

### Study cohort and sample collection

Prior to study, the medical ethics approval was obtained from the People’s Hospital of Guangxi Zhuang Autonomous Region (No.KY-DZX202008) and written informed consent was obtained by each subject. The study was carried out in accordance with the Declaration of Helsinki. For serum lipidomics analysis, a total of 100 subjects including 50 NR and 50 CA subjects were enrolled in this study. At the same time, we evaluated for possible sex and age-associated differences by Chi-square test and Student’s test. Detailed characteristics of the study cohort were shown in Table 1. All whole-blood samples were taken after an 8-h fast, left to stand at room temperature for 25 min, and serum was then collected following centrifugation at 5000 rpm/min for 10 min at 4 °C. The serum samples were immediately stored at-80 °C prior to analysis.

**Table 1:** Information of clinical characteristics for study cohort

Group	Gender (Female/Male)	Age (year)	Position	Vienna classification
**NR** (*n* = 50)	21/29	53 ± 8	–	–
**CA** (*n* = 50)	18/32	56 ± 12	Rectum (23)	High (26)
			Colon (27)	Low (24)
***P* vaule**	0.682	0.238	–	–

The statistical analysis for composition of gender and age between NR and CA groups was conducted by Chi-square test and Student’s test, respectively

*Abbreviations*: *CA* Colorectal adenoma, *NR* Normal control

### Sample preparation for lipidomic analysis

For sample preparation, 50 μL serum sample was added and mixed with 500 μL precooling dichloromethane-methanol (3:1, v/v) solution. After vortexed for 5 min and placed in ice bath for 10 min, the solution was centrifuged at 13,000 rpm/min at 4 °C for 10 min. 300 μL lower dichloromethane solution was dried in vacuum at room temperature. The dried samples were redissolved with 600 μL acetonitrile-isopropanol (1:1, v/v) solution, then vortexed for 2 min and ultrasonicated in ice bath for 5 min. Next vortexed for 1 min, the mixture was centrifuged at 13,000 rpm/min at 4 °C for 15 min, and the supernatant was used for serum lipid analysis. Quality control (QC) samples were prepared by mixing 5 μL of each sample to ensure the stability and reproducibility of data acquisition.

### UHPLC–ESI-HRMS-based lipidomic analysis

A Dionex Ultimate 3000 liquid chromatography system (Sunnyvale, CA, USA) (SN: 7254012) coupled to a Thermo Fisher Q Exactive Orbitrap mass spectrometry system (Waltham, MA, USA) (SN: SN02386L) were used for lipidomics analysis. The LC conditions were as follows: column, Waters Acquity UPLC HSS T3 (1.8 μm, 2.1 × 100 mm; Milford, MA, USA); mobile phase A, acetonitrile-water (60:40, v/v) containing 0.1% formic acid and 10 mM ammonium formate; mobile phase B, isopropanol-acetonitrile (90:10, v/v) containing 0.1% formic acid and 10 mM ammonium format; The gradient conditions were set as follows: 0.0–4.0 min, 30 to 60% B; 4.0–9.0 min, 60 to 100% B; 9.0–15.0 min, 100% B; 15.0–18.0 min, 100% B to 30% B. The injection volume was 5 μL, and the column temperature was 50 °C, as well as the flow rate was 0.3 mL/min.

The MS spectrometric parameters were as follows: spray voltage, 3.5 kV; sheath gas flow rate, 50 psi; auxiliary gas flow rate, 13 arb; capillary temperature, 320 °C; auxiliary gas heater temperature, 420 °C; scan modes, full MS (resolution 70,000) and ddMS2 (resolution 17,500 with stepped collision energy (10, 20, and 40 eV); and scan range, *m/z* 100–1200. All data were acquired using the Thermo Scientific Xcalibur 3.1 software (Waltham, MA, USA).

### Statistical analysis

Univariate statistical analysis: Raw data files were imported into the Compound Discoverer™3.1 (Thermo Scientific, Fremont, CA, USA) for data analysis. Lipidomics data (including all ion features with their RT, *m/z*, and peak intensity) were extracted and normalization was conducted by using QC samples to effectively uncover differential lipids. The feature differences between groups were analyzed with Mann-Whitney *U* test or Student’ *t*-test based on distribution characteristics of the data. The value of *P* < 0.05 was considered to indicate significant differences. A list of potential lipids was identified depending on Thermo mzVault and LipidBlast database. The main parameters were as follows: minimum peak intensity, 500,000, mass error, 10 ppm, RT tolerance, 0.2 min, intensity tolerance, 30%, S/N, 3.

Multivariate statistical analysis: Principal component analysis (PCA) and orthogonal partial least squares discriminant analysis (OPLS-DA) were performed with the software SIMCA-P 14.1 (Umetrics, UMEA, Sweden). To avoid overfitting, 200 times permutation test was carried out on the analytical model. The criteria of fold change > 1.5 or < 0.67 and *P* < 0.05 were set as the cut-off values for selection of differential lipids between groups. The ROC analysis of the differential lipids was performed by MetaboAnalyst 5.0 (https://www.metaboanalyst.ca/) to evaluate the diagnostic performance and to explore the potential biomarkers for CA patients.

## Results

### Differential lipid profiles between CA and NR

**Fig. 1:**
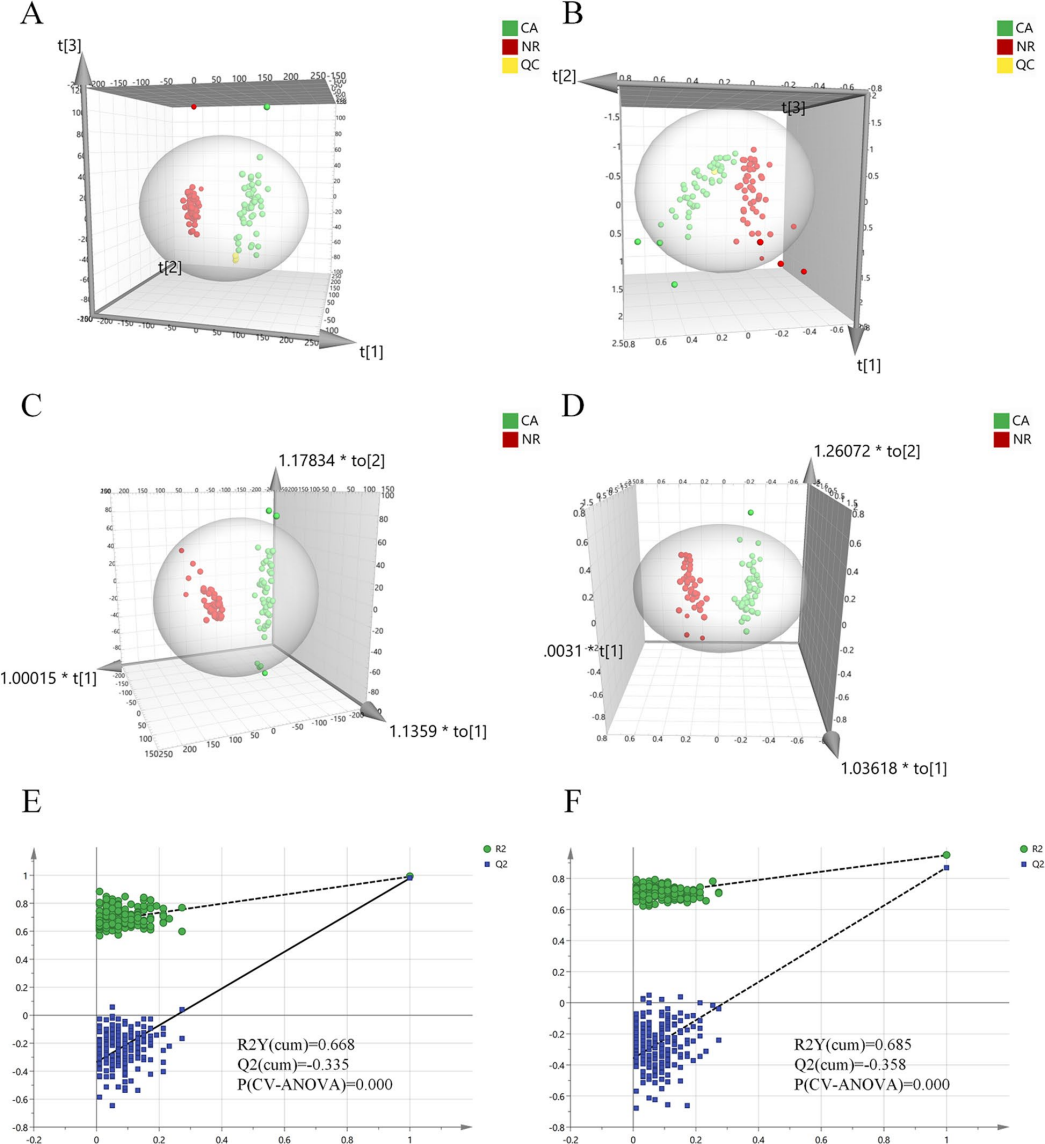
Multivariate statistical analysis of differential lipid features between NR and CA groups in both ESI modes. Principal component analysis (PCA) of two groups in ESI+ and ESI- modes, respectively (**A**, **B**); orthogonal partial least squares discriminant analysis (OPLS-DA) of the two groups in ESI+ and ESI- modes, respectively (**C**, **D**); Overfitting test for OPLS-DA model in ESI+ and ESI- modes, respectively (**E**, **F**). Abbreviations: ESI, electrospray ionization; CA, colorectal adenoma; NR, normal control

PCA and OPLS-DA models, the common multivariate statistical methods used in omics study, were utilized to evaluate the differences between groups regarding lipid metabolism of CA and NR groups. Firstly, the PCA model was constructed and its score plots performed on all the samples revealed that the QC samples were clustered closely in both ESI modes, indicating the analysis system with excellent robustness and reproducibility during the batch analysis process (Fig. 1A and B). In addition, most samples contained in the 95% confidence interval apart from a few exceptions. It could be considered individual variations for a few samples outside the confidence interval (Fig. 1A and B). The relatively smaller individual difference of lipid profiles in NR patients was seen compared with the CA patients, which could be due to the pathogenic factors. In addition, a distinct separation between the two groups presented in two modes also reflected their differences in lipid metabolism (Fig. 1A and B). Meanwhile, Relative to the ESI- mode, the ESI+ mode had more obvious separation trend (Fig. 1A and B). To evaluate the effect of gender and age on the difference in lipid profiles between two groups, statistical analysis was conducted and the results showed no significant difference between CA and NR groups for the gender and age in study cohort (Table 1).

Furthermore, to maximize the discovery of differential lipid features in the serum between CA and NR groups, the OPLS-DA model was established using all of detected lipid features. Results showed that two groups were clearly discriminated at ESI+ mode (R2X [cum] = 0.386, R2Y [cum] = 0.962, Q2 [cum] = 0.956) and ESI- mode (R2X [cum] = 0.492, R2Y [cum] = 0.945, Q2 [cum] = 0.890), respectively, which indicated remarkable differences between groups in both ESI modes and obvious dysregulation in lipid metabolism of CA relative to NR group (Fig. 1C and D). Meanwhile, a 200 times permutation test was performed to verify the reliability and applicability of OPLS-DA model for data analysis. The intercept of the R2Y and Q2 was 0.668 and − 0.355 in ESI+ mode, and R2Y and Q2 was 0.685 and − 0.358 in ESI- mode, and value of *P* (CV-ANOVA) was 0.000 in both modes, respectively (Fig. 1E and F). Concurrently, R2Y and Q2 values derived from the permutation test were all lower than corresponding original values, which provided proof that OPLS-DA model was rational and not overfitting for the data analysis in both ESI modes.

### Screening and identification of diagnostic lipid biomarkers for CA

**Fig. 2:**
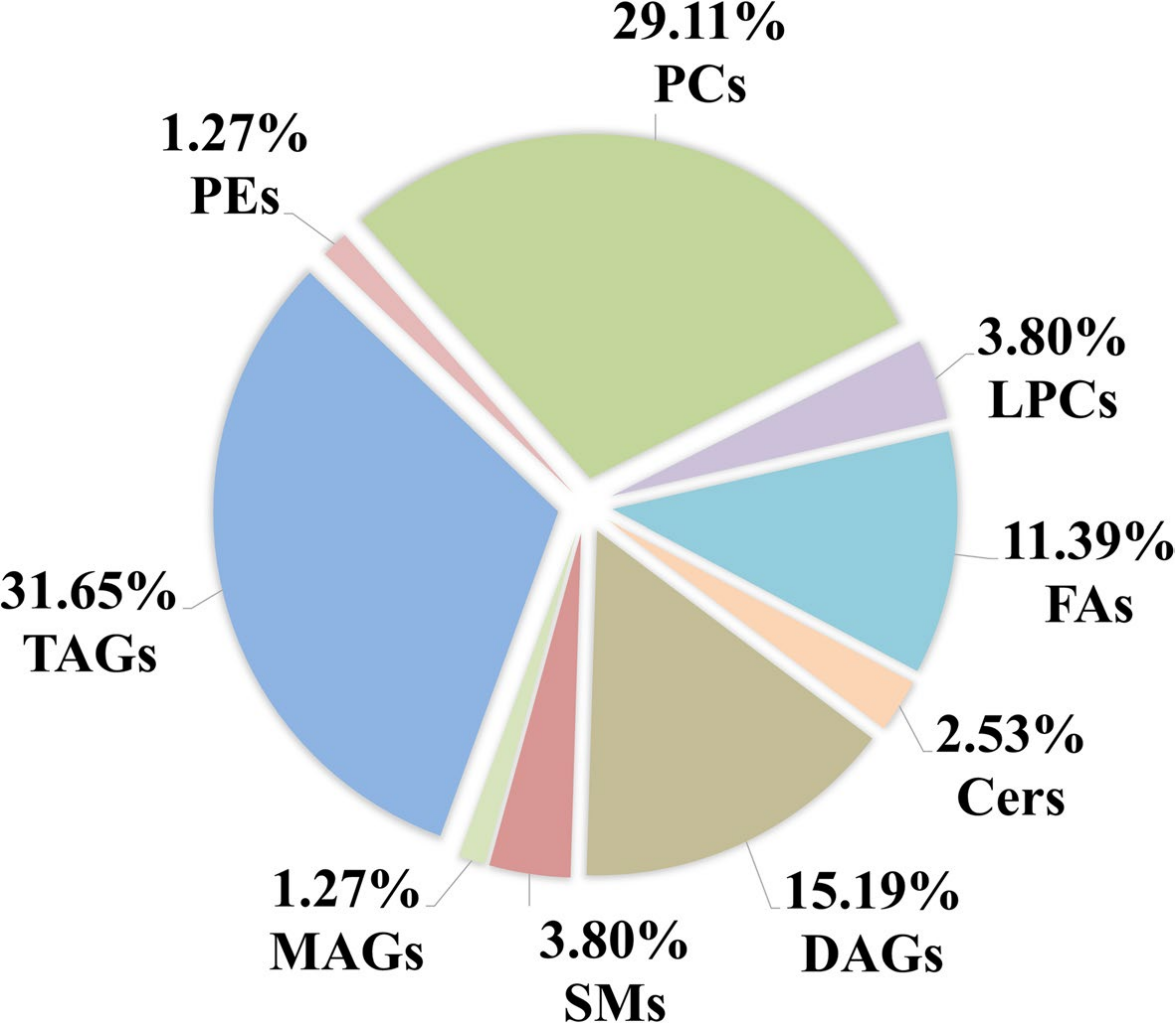
The constituent ratio of differential lipids in serum between CA and NR groups. Abbreviations: CA, colorectal adenoma; NR, normal control; MAGs, monoacylglycerols; DAGs, diacylglycerols; TAGs, triacylglycerols; LPCs, lysophosphatidylcholines; PEs, phosphatidylethanolamines; Cers, ceramides; PCs, phosphatidylcholines; SMs, sphingomyelins; FAs, fatty acids

**Fig. 3:**
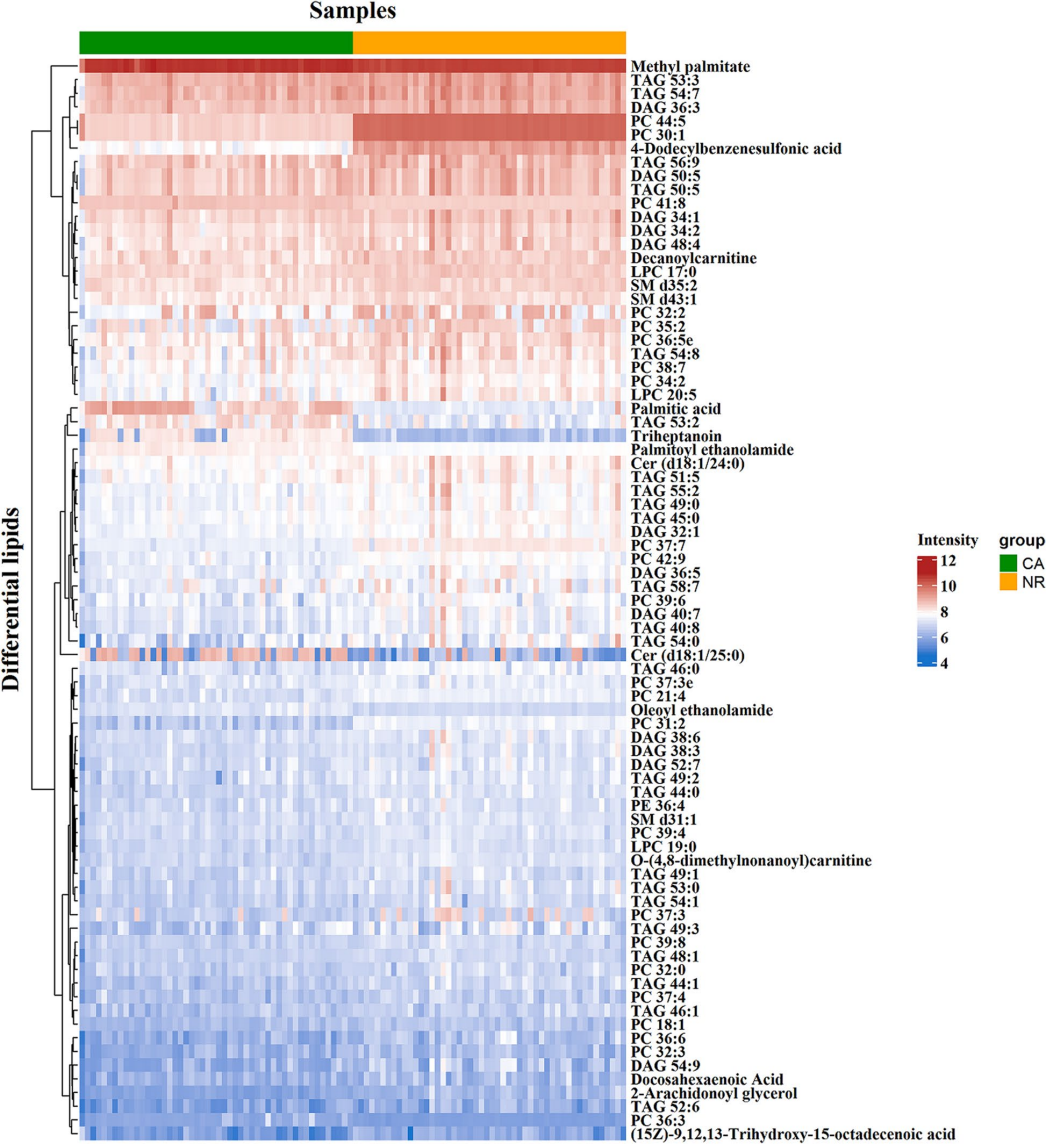
Level distribution of differential lipids between CA and NR groups. Clustering heatmap was draw using R software by data transforming with log10. The majority of differential lipids in the CA group showed a tendency of significant decrease compared to NR group. Abbreviations: CA, colorectal adenoma; NR, normal control; DAG, diacylglycerol; TAG, triacylglycerol; LPC, lysophosphatidylcholine; PE, phosphatidylethanolamine; Cer, ceramide; PC, phosphatidylcholine; SM, sphingomyelin

The difference analysis in serum lipid profiles between NR and CA groups was performed using the univariate and multivariate statistical methods. To minimize false positives, combined with fold change > 1.5 or < 0.67 and *P* < 0.05, finally, a total of 79 differential lipids were found between the groups, including 4 in ESI- mode and 75 in ESI+ mode (Table 2). These differential lipids mainly included monoacylglycerols (MAGs): 1.27%, diacylglycerols (DAGs): 15.19%, TAGs: 31.65%, FAs: 11.39%, LPCs: 3.80%, PCs: 29.11%, phosphatidylethanolamines (PEs): 1.27%, ceramides (Cers): 2.53% and sphingomyelins (SMs): 3.80% (Fig. 2). TAGs and PCs made up the highest fraction in differential lipid types, followed by DAGs and then FAs. Therefore, the two lipid types accounted for 60.76% of the total proportion, suggesting that dysregulation of PC and TAG metabolism is closely associated with the diagnosis and pathogenesis of CA disease. Furthermore, to learn more about the distribution of relative levels of differential lipids in two groups, the identified lipid data were analyzed using clustering heatmap. Just showed the Table 2 and Fig. 3, the majority of differential lipids features were significantly down-regulated in CA group compared to NR group. Taken together, PCs and TAGs are considered to be the main influencing factor that contributed to the CA formation.

**Table 2:** Differential lipids between NR and CA groups in both ESI modes

Name	Formula	Scan mode	Detective ***m/z***	RT (min)	***P*** value	Fold change	AUC
Triheptanoin	C24 H44 O6	ESI+	451.30408	5.32	0.000	30.57	0.850
TAG 58:7	C61 H104 O6		950.81793	12.64	0.024	0.44	0.665
TAG 55:2	C58 H108 O6		914.84888	14.23	0.010	0.27	0.756
TAG 54:8	C57 H94 O6		892.73761	11.49	0.029	0.46	0.674
TAG 54:7	C57 H96 O6		894.74548	11.76	0.001	0.53	0.725
TAG 54:1	C57 H108 O6		906.84503	14.32	0.001	0.38	0.791
TAG 54:0	C57 H110 O6		908.86298	14.54	0.020	0.28	0.705
TAG 53:2	C56 H104 O6		890.80969	13.09	0.000	4.02	0.862
TAG 53:0	C56 H108 O6		894.84143	14.24	0.032	0.20	0.778
TAG 52:6	C55 H94 O6		873.69482	11.36	0.000	0.39	0.703
TAG 51:5	C54 H94 O6		856.73828	11.67	0.006	0.57	0.668
TAG 50:5	C53 H92 O6		842.72321	11.48	0.005	0.50	0.698
TAG 49:3	C52 H94 O6		832.73969	12.17	0.006	0.38	0.664
TAG 49:2	C52 H96 O6		834.74469	12.07	0.001	0.60	0.728
TAG 49:1	C52 H98 O6		836.77118	12.75	0.032	0.40	0.704
TAG 49:0	C52 H100 O6		838.78638	13.55	0.003	0.39	0.745
TAG 48:1	C51 H96 O6		822.75482	12.36	0.000	0.60	0.809
TAG 46:1	C49 H92 O6		794.72369	11.88	0.000	0.50	0.752
TAG 46:0	C49 H94 O6		796.74139	12.42	0.000	0.41	0.815
TAG 45:0	C48 H92 O6		782.71979	12.11	0.000	0.55	0.780
TAG 44:1	C47 H88 O6		766.69427	11.31	0.000	0.33	0.824
TAG 44:0	C47 H90 O6		768.70270	11.68	0.000	0.42	0.860
SM d43:1	C48 H97 N2 O6 P		829.71655	10.61	0.000	0.66	0.752
SM d35:2	C40 H79 N2 O6 P		715.57685	8.47	0.000	0.63	0.787
SM d31:1	C36 H73 N2 O6 P		661.52917	7.66	0.000	0.64	0.747
PC 36:5e	C44 H80 N O7 P		766.57556	8.67	0.006	0.57	0.718
PC 44:5	C52 H94 N O8 P		892.67596	10.43	0.000	0.03	1.000
PC 39:8	C47 H78 N O8 P		816.55990	8.55	0.001	0.55	0.760
PC 37:3	C45 H84 N O8 P		798.60315	9.31	0.003	0.22	0.709
PC 32:2	C40 H76 N O8 P		730.53705	8.50	0.004	0.49	0.616
PC 21:4	C29 H50 N O8 P		572.33582	2.70	0.000	0.47	0.900
PC 34:2	C42 H80 N O8 P		758.57068	8.60	0.001	0.56	0.744
Palmitoyl ethanolamide	C18 H37 N O2		300.28989	4.32	0.000	2.02	0.973
Palmitic acid	C16 H32 O2		274.27435	1.19	0.000	17.64	0.930
Oleoyl ethanolamide	C20 H39 N O2		326.30597	4.71	0.000	2.01	0.883
O-(4,8-dimethylnonanoyl)carnitine	C18 H35 N O4		330.26450	1.04	0.000	0.47	0.872
Methyl palmitate	C17 H34 O2		288.29022	1.35	0.000	1.52	0.943
LPC 20:5	C28 H48 N O7 P		542.32483	1.95	0.046	0.35	0.772
PC 32:0	C40 H80 N O8 P		734.57001	9.07	0.005	0.47	0.712
DAG 38:6	C41 H68 O5		641.51538	9.23	0.015	0.33	0.758
DAG 36:5	C39 H66 O5		615.49921	8.99	0.001	0.40	0.777
DAG 34:2	C37 H68 O5		609.59497	9.67	0.002	0.50	0.742
Decanoylcarnitine	C17 H33 N O4		316.24884	0.97	0.000	0.62	0.781
Cer (d18:1/25:0)	C43 H85 N O3		664.66088	11.05	0.001	2.50	0.661
Cer (d18:1/24:0)	C42 H83 N O3		650.64496	10.84	0.002	0.40	0.779
DAG 38:3	C41 H74 O5		647.56055	10.11	0.017	0.32	0.777
2-Arachidonoyl glycerol	C23 H38 O4		379.28247	5.25	0.000	0.24	0.933
TAG 56:9	C59 H96 O6		918.75745	11.46	0.003	0.56	0.686
PC 32:3	C40 H74 N O8 P		728.52399	7.89	0.001	0.51	0.712
DAG 32:1	C35 H66 O5		584.52533	9.58	0.000	0.47	0.816
PC 31:2	C39 H74 N O8 P		716.56000	8.60	0.000	0.18	0.993
DAG 34:1	C37 H70 O5		612.55823	10.01	0.002	0.46	0.745
DAG 36:3	C39 H70 O5		636.55676	9.69	0.002	0.50	0.759
PC 36:3	C44 H82 N O8 P		784.58606	8.13	0.019	3.49	0.950
DAG 40:7	C43 H70 O5		667.53107	9.45	0.017	0.31	0.730
PC 30:1	C38 H74 N O8 P		704.52200	8.57	0.000	0.03	1.000
PE 36:4	C41 H74 N O8 P		740.52350	8.75	0.013	0.51	0.738
LPC 17:0	C25 H52 N O7 P		510.35626	4.22	0.000	0.63	0.822
DAG 54:9	C59 H90 O6		895.67676	11.11	0.001	0.18	0.753
DAG 52:7	C57 H90 O6		871.67627	11.35	0.004	0.46	0.703
DAG 48:4	C51 H90 O6		816.70032	11.38	0.005	0.48	0.680
DAG 50:5	C53 H92 O6		842.72473	11.46	0.004	0.50	0.696
PC 35:2	C43 H82 N O8 P		772.58594	8.97	0.000	0.46	0.777
PC 36:6	C44 H76 N O8 P		778.53882	7.52	0.002	0.21	0.784
PC 41:8	C49 H82 N O8 P		844.62268	9.07	0.000	1.65	0.990
PC 18:1	C60 H94 O16		536.33563	1.10	0.000	0.61	0.739
TAG 53:3	C56 H102 O6		888.80316	12.97	0.001	0.56	0.724
TAG 40:8	C43 H68 O5		665.51434	9.04	0.004	0.40	0.734
PC 39:4	C47 H86 N O8 P		824.61963	9.37	0.000	0.64	0.756
PC 37:7	C45 H76 N O8 P		790.53979	7.81	0.000	0.21	0.977
PC 38:7	C46 H78 N O8 P		804.55463	7.89	0.049	0.50	0.699
PC 42:9	C50 H82 N O8 P		856.58630	8.22	0.000	0.48	0.881
LPC 19:0	C27 H56 N O7 P		538.38757	5.25	0.000	0.55	0.824
PC 39:6	C47 H82 N O8 P		820.58752	8.74	0.000	0.55	0.699
PC 37:3e	C45 H86 N O7 P		784.58527	9.39	0.008	0.64	0.714
PC 37:4	C46 H84 N O10 P	ESI-	840.57782	8.93	0.001	0.54	0.707
Docosahexaenoic acid	C22 H32 O2		327.23349	4.67	0.015	0.41	0.620
4-Dodecylbenzenesulfonic acid	C18 H30 O3 S		325.18488	2.64	0.000	0.04	1.000
(15Z)-9,12,13-Trihydroxy-15-Octadecenoic acid	C18 H34 O5		329.23358	0.92	0.012	0.54	0.765

Fold change: the arithmetic mean values of peak intensity of CA/NR

*Abbreviations*: *AUC* Area under the curve, *RT* Retention time, *m/z* Mass to charge ratio, *DAG* Diacylglycerol, *TAG* Triacylglycerol, *LPC* Lysophosphatidylcholine, *PC* Phosphatidylcholine, *PE* Phosphatidylethanolamine, *Cer* Ceramide, *SM* sphingomyelin

### Performance evaluation and verification of potential lipid biomarkers of CA

**Fig. 4:**
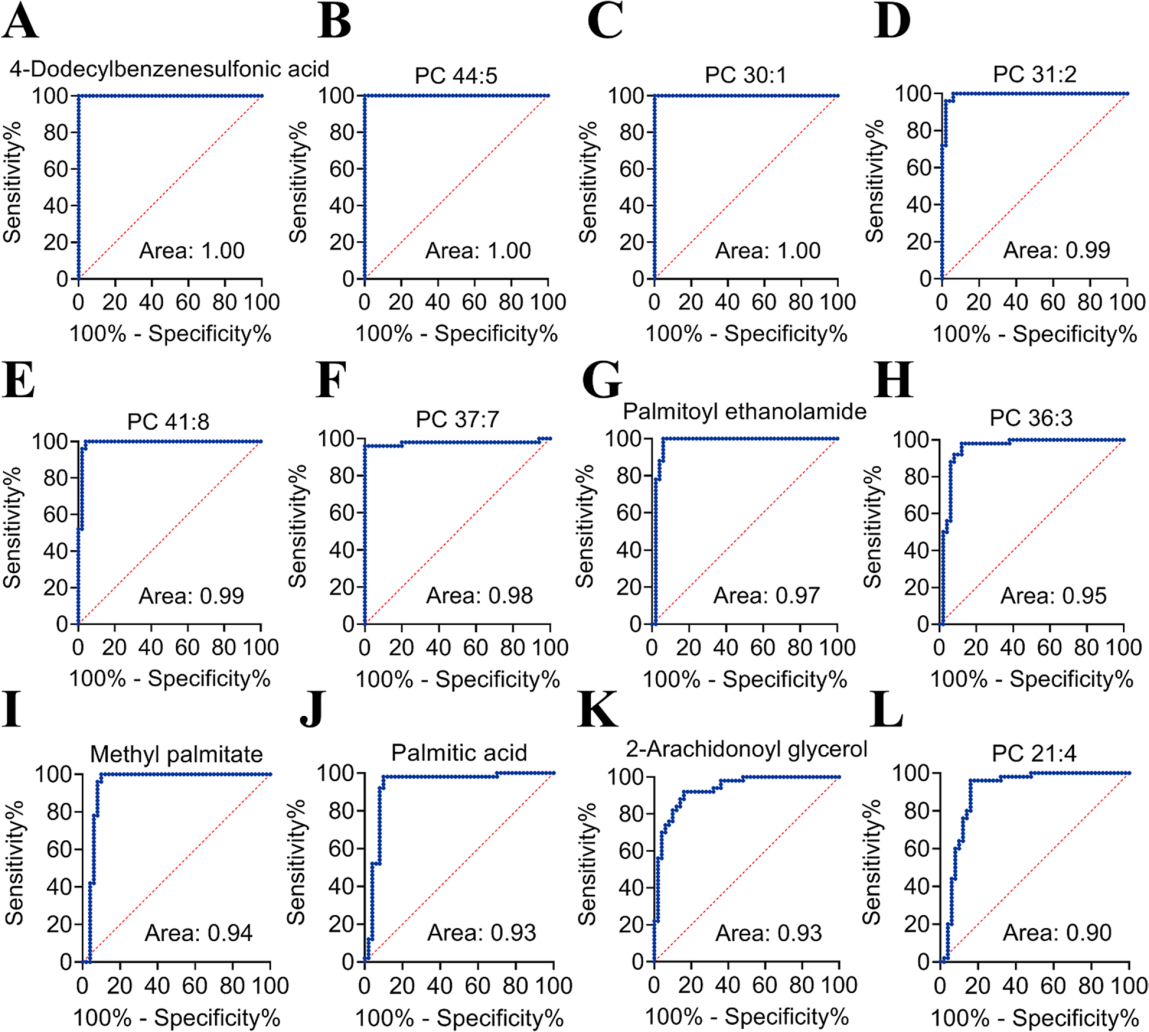
Performance evaluation of differential lipids between CA and NR groups. Potential lipid biomarkers for CA diagnosis were selected based on the AUC value more than 0.900. Abbreviations: CA, colorectal adenoma; NR, normal control; AUC, area under the curve; PC, phosphatidylcholine

**Fig. 5:**
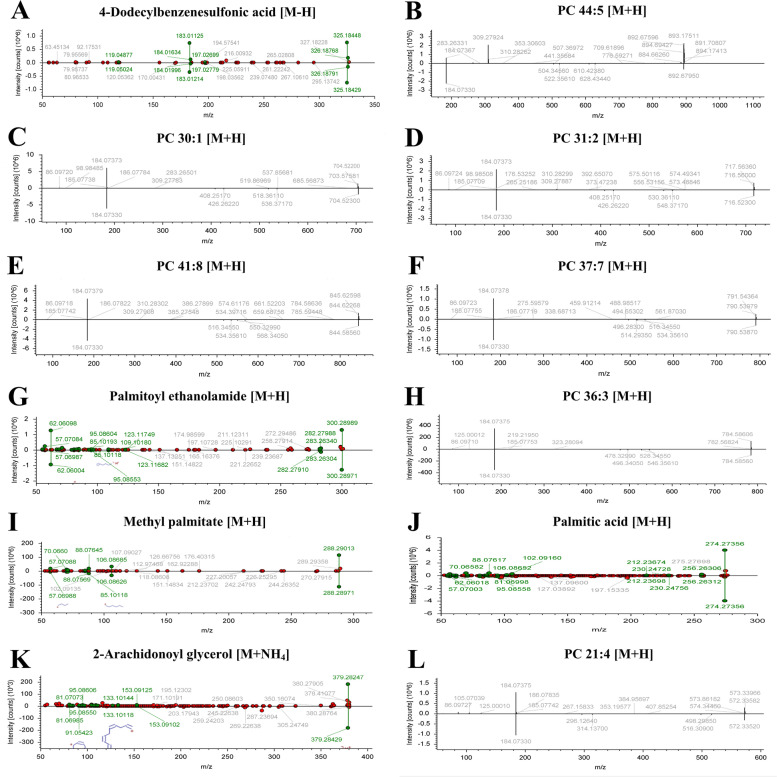
The identification of differential lipids with high discriminate ability (AUC ≥ 0.900) for the CA and NR groups. Abbreviations: CA, colorectal adenoma; NR, normal control; AUC, area under the curve; PC, phosphatidylcholine

**Fig. 6:**
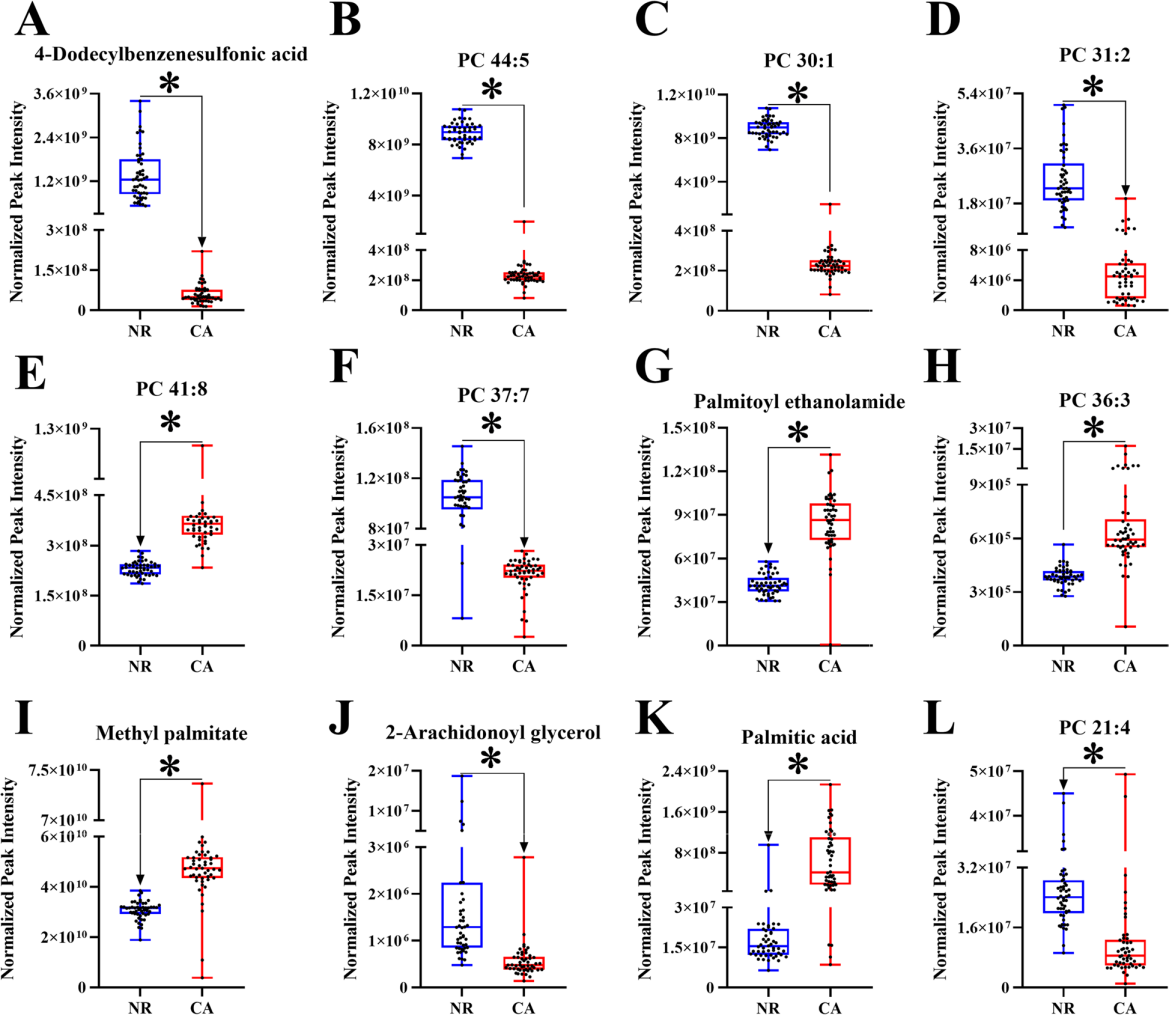
The change trend of 12 differential lipids with high performance for CA diagnosis between two groups. The levels of differential lipids between CA and NR groups were displayed with mean ± SEM. The “★” represented statistical significance of the variate with *P* < 0.05 between two groups. Abbreviations: CA, colorectal adenoma; NR, normal control; AUC, area under the curve; PC, phosphatidylcholine; SEM, standard error of mean

**Fig. 7:**
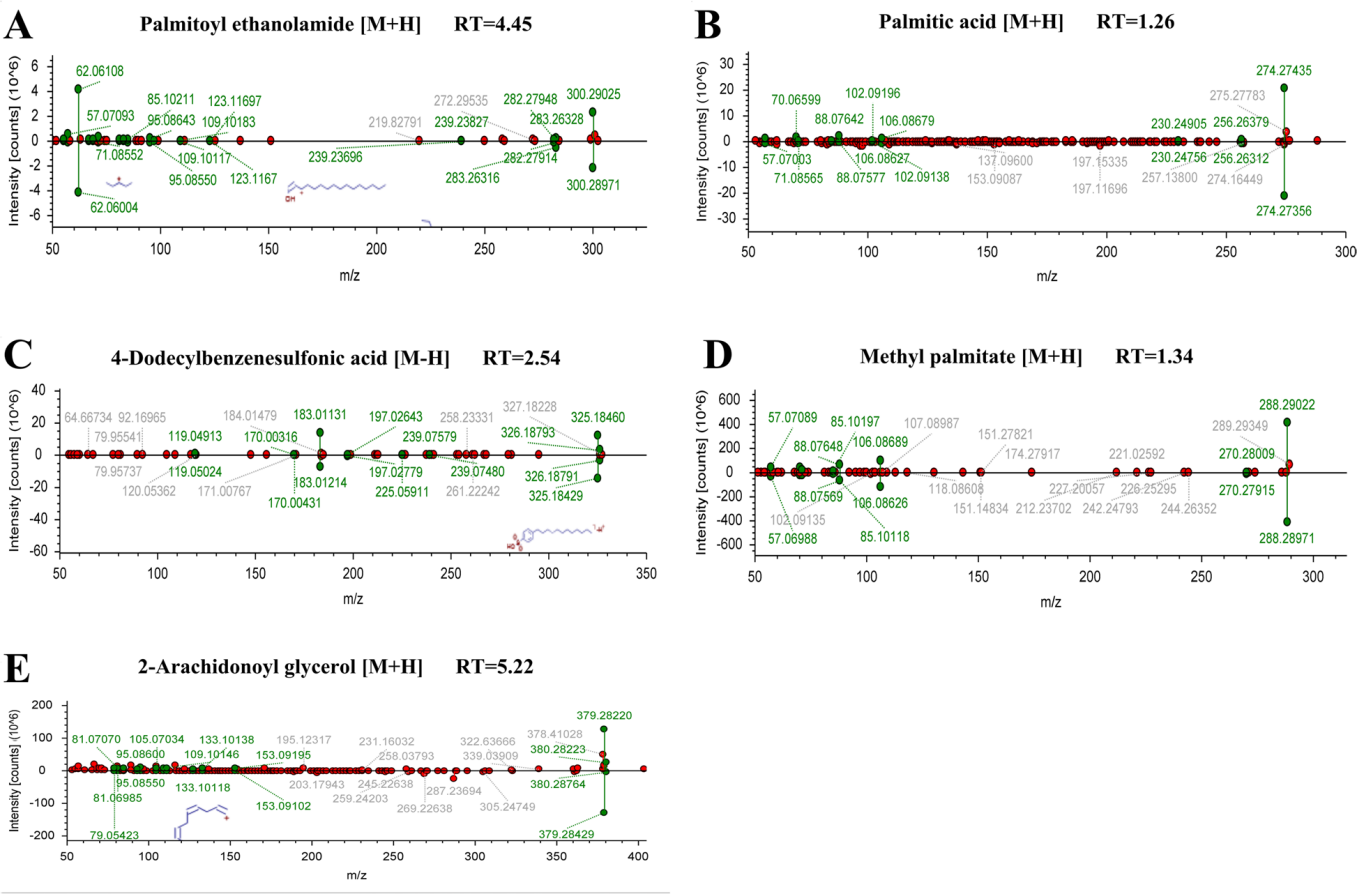
Verification of potential lipid biomarkers with high performance for CA diagnosis by lipid standards. Abbreviations: CA, colorectal adenoma

The diagnostic performance of 79 differential lipids between CA and NR was evaluated by ROC analysis using MetaboAnalyst 5.0, which could maximize the area under the curve (AUC) as calculated by the trapezoidal method to select the most suitable cut-off point. Before performing ROC analysis, sum normalization and auto-scaling were carried out for lipidomic data to effectively reduce the influence of individual differences and systematic errors. Generally, the AUC values of the differential lipids ranged from 0.616 to 1.000, and most of them had comparatively low AUC values (Table 2). By combining with AUC ≥ 0.900 as selected criteria, we obtained 12 differential lipids with good diagnostic performance for CA (Fig. 4), mainly including 7 PCs, 4 FAs lipids, and 1 MAG, and which were identified by matching the high resolution MS, MS/MS fragments, and RT from Thermo mzCloud and mzVault with Lipidblast databases (Fig. 5). Among them, PC 30:1, PC 44:5 and 4-dodecylbenzenesulfonic acid had the highest AUC values (1.000 (95% CI: 1.000–1.000)), indicating outstanding diagnostic ability for CA (Fig. 4), while PC 21:4 had the relatively low AUC value (0.900 (95% CI: 0.830–0.969)). Based on the ROC analysis, we further explored the change trend of levels for these 12 potential lipid biomarkers with good distinguish efficacy between two groups. The results showed that five lipids including PC 41:8, PC 36:3, palmitoyl ethanolamide, methyl palmitate, and palmitic acid were significantly up-regulated in the CA group, while the remaining seven lipids including 4-dodecylbenzenesulfonic acid, PC 44:5, PC 30:1, PC 31:2, PC 37:7, PC 21:4, and 2-arachidonoyl glycerol were remarkably down-regulated in the CA group compared with NR group (Fig. 6). Among them, PC 44:5, PC 30:1, palmitic acid and 4-dodecylbenzenesulfonic acid presented the most significant change trend with the fold change more than 10 (Table 2), meanwhile, which was consistent with the clustering heatmap of differential lipids between groups. Additionally, to further confirm the potential lipid biomarkers, we applied the commercial lipid standards to verify the potential lipid biomarkers of CA by matching the exact mass, retention time and fragmentation pattern under the same LC-MS conditions for lipidomic study (Fig. 7). Hence, the above 5 differential lipids identified with lipid standards served as the potential diagnostic biomarkers for CA.

## Discussion

In this study, serum lipid profiles were found to be distinctly different between the NR and CA groups. After an efficient and robust analysis, a total of 79 differential lipids were found between groups. Among them, TAGs and PCs were the main lipid types, disclosing the metabolic perturbation of TAGs and PCs could be involved in the CA formation. Furthermore, 12 differential lipids showed good diagnostic performance as the potential biomarkers for CA. PCs and FAs are the main dysregulated lipid biomarkers, particularly, three lipids of PC 30:1, PC 44:5, and 4-dodecylbenzenesulfonic acid with outstanding diagnostic ability for CA.

Over the past decade, although some research has overwhelmingly focused on the discovery of potential diagnostic biomarkers of CRC, few studies have committed to exploring the lipid markers for CA diagnosis. Rachieriu C et al. analyzed the serum lipid profiles of CRC patients by HPLC-QTOF-MS, and found 25 potential markers with AUC values > 0.750, principally including PCs, Cers, FAs [26]. Similarly, plasma lipidomics showed that PG 34:0, SM 42:2, Cer 44:5, LPC 18:3, LPC 18:2, O-PE 36:3, O-PE 38:3 and SM 38:8 with good performance (AUC > 0.800) could act as promising diagnostic biomarkers for CRC screening [18]. Moreover, the combination of LPC 17:0, LPC 19:0, LPC 19:1 and LPC 19:2 could better distinguish between NR and CRC patients (AUC = 0.863) through a targeted lipidomic study [27]. As the severe stage of CA, colorectal advanced adenoma (CAA) was also considered as the effective target for CRC prevention. In our recent study, we disclosed that triglyceride (TAG) was the major dysregulated lipids in CAA, and 12 differential lipids served as the potential biomarkers of CAA diagnosis [28]. In addition to lipidomics, metabolomics is also used extensively in the discovery of lipid biomarkers for CA or CRC. Another study based on stool metabolomics showed that combination of ChoE 18:1, ChoE 18:2, ChoE 20:4, PE 16:0/18:1, SM d18:1/23:0, SM 42:3, and TAG 54:1 could effectively discriminate NR and CRC as the diagnostic biomarker, and its integrated performance was good (AUC = 0.821) [29]. In the plasma metabolomics, 48 differential metabolites were uncovered between CA and CRC, mainly including LPCs and PCs, and both of them were down-regulated in CRC [30]. In tissue metabolomics, PC 32:1 was suggested as an invaluable biomarker, which could be used for clinical diagnosis for CRC by imaging mass spectrometry [31]. PCs are the important lipid carrier in plasma, and phospholipids related to choline were considered good biomarkers of CRC [32, 33]. Furthermore, the most abundant metabolic features identified in the CA patients were PCs and PEs, and LPC (P-16:1) could be a putatively novel lipid signature [24]. The disorder of PC metabolism was believed to be strongly linked with the risk of CA [34]. Similarly, our previous study reported that metabolism of linoleic acid and phospholipid exhibited remarkable dysregulation in the CA patients by plasma metabolomics [22]. In this study, the PCs also acted as the main potential lipid markers for CA diagnosis (Figs. 2 and 4).

Apart from PCs, FAs are also potential diagnostic markers for distinguishing CRC or CA from NR according to the previous reports [19, 35]. Altered plasma levels of decanoic acid in CRC could serve as a new diagnostic biomarker [36]. Studies have noted a rise in the level of total TAGs in serum or plasma may be related to the elevated risk of CA [23]. Similar result that TAGs were the main dysregulated lipids in the CA group was observed in our study (Fig. 2). TAGs storage in adipose tissue is the major reservoir for energy metabolism in mammals. During lipolysis, FAs are hydrolyzed from TAGs stores and then transported to other tissues for catabolism [37]. So, the perturbation of TAG metabolism generally dysregulates the FA metabolism. Furthermore, FAs as essential components of biological membranes. It has been found that many cancer cells express higher levels of FAs than corresponding normal cells because cancer cells require substantial lipids for energy synthesis, signal transduction, and more membranes for vigorous metabolism [38, 39]. However, although few FAs including triheptanoin, palmitoyl ethanolamide, palmitic acid, oleoyl ethanolamide, methyl palmitate had increase levels in CA group, most of FAs presented significant down-regulation in CA (Table 2 and Fig. 3). Additionally, the fecal metabolome results found the level of palmitoyl ethanolamide in CA group was visibly higher than NR group, which could serve as putative biomarker of CA [25]. For serum metabolomics [40], the level of palmitic acid in CRC patients showed a noticeable up-regulation trend, meanwhile, which also exhibited significant increase with fold change of 17.64 in CA (Table 2 and Fig. 3), indicating which could be a candidate biomarker of CRC progression. In ROC analysis, the 12 potential lipid biomarkers presented good diagnostic performance for CA screening (AUC ≥ 0.900), containing 7 PCs, 4 FAs, and 1 MAG (Fig. 4), which may contribute to the early discovery and prevention of CRC. In summary, the perturbation of PCs and TAGs metabolism may be closely relevant to CA formation, and the PCs and FAs are the major dysregulated potential biomarkers for CA diagnosis. These discoveries should provide a valuable reference for the early screening and carcinogenesis of CRC.

## Conclusions

To our knowledge, the present study is the first to explore lipid biomarkers for CA based on serum lipidomics with large-scale samples. In this research, obvious differences in the serum lipid profiles between CA and NR were observed, and 79 differential lipids were selected by UHPLC-ESI-HRMS-based serum lipidomics. TAGs and PCs were predominant components of differential lipids, indicating the abnormal metabolism of them should contribute to the formation of CA. In addition, 12 serum differential lipids were verified as the potential biomarkers for CA screening owing to their excellent diagnostic performance. Generally, this study provides a novel light into the lipid metabolism pathways associated with CA formation. Simultaneously, the discovery of lipid biomarkers for CA may also offer new insights for its clinical diagnosis. Due to limitations in sample sizes and study models, undoubtedly, further validation is needed for our discovery.

## Data Availability

The datasets supporting the conclusions of this article are included within the article.

## Abbreviations

UHPLC: Ultra high performance liquid chromatography
ESI: Electrospray ionization
HRMS: High resolution mass spectrometry
CA: Colorectal adenoma
NR: Normal control
CRC: Colorectal cancer
AUC: Area under the curve
RT: Retention time
*m/z*: Mass to charge ratio
MAGs: Monoacylglycerols
DAGs: Diacylglycerols
TAGs: Triacylglycerols
LPCs: Lysophosphatidylcholines
PEs: Phosphatidylethanolamines
Cers: Ceramides
PCs: Phosphatidylcholines
SMs: Sphingomyelins
FAs: Fatty acids
